# Occupational Exposure to Polychlorinated Biphenyls and Risk of Breast Cancer

**DOI:** 10.1289/ehp.11774

**Published:** 2008-09-26

**Authors:** Sharon R. Silver, Elizabeth A. Whelan, James A. Deddens, N. Kyle Steenland, Nancy B. Hopf, Martha A. Waters, Avima M. Ruder, Mary M. Prince, Lee C. Yong, Misty J. Hein, Elizabeth M. Ward

**Affiliations:** 1 National Institute for Occupational Safety and Health, Division of Surveillance, Hazard Evaluations and Field Studies, Cincinnati, Ohio, USA; 2 Department of Epidemiology, Emory University School of Public Health, Atlanta, Georgia, USA; 3 Department of Environmental Health, University of Cincinnati, Cincinnati, Ohio, USA; 4 Sanofi-aventis, Bridgewater, New Jersey, USA; 5 American Cancer Society, Atlanta, Georgia, USA

**Keywords:** breast cancer, incidence, occupational epidemiology, polychlorinated biphenyls

## Abstract

**Background:**

Despite the endocrine system activity exhibited by polychlorinated biphenyls (PCBs), recent studies have shown little association between PCB exposure and breast cancer mortality.

**Objectives:**

To further evaluate the relation between PCB exposure and breast cancer risk, we studied incidence, a more sensitive end point than mortality, in an occupational cohort.

**Methods:**

We followed 5,752 women employed for at least 1 year in one of three capacitor manufacturing facilities, identifying cases from questionnaires, cancer registries, and death certificates through 1998. We collected lifestyle and reproductive information via questionnaire from participants or next of kin and used semiquantitative job-exposure matrices for inhalation and dermal exposures combined. We generated standardized incidence ratios (SIRs) and standardized rate ratios and used Cox proportional hazards regression models to evaluate potential confounders and effect modifiers.

**Results:**

Overall, the breast cancer SIR was 0.81 (95% confidence interval, 0.72–0.92; *n* = 257), and regression modeling showed little effect of employment duration or cumulative exposure. However, for the 362 women of questionnaire-identified races other than white, we observed positive, statistically significant associations with employment duration and cumulative exposure; only smoking, birth cohort, and self- or proxy questionnaire completion had statistically significant explanatory power when added to models with exposure metrics.

**Conclusions:**

We found no overall elevation in breast cancer risk after occupational exposure to PCBs. However, the exposure-related risk elevations seen among nonwhite workers, although of limited interpretability given the small number of cases, warrant further investigation, because the usual reproductive risk factors accounted for little of the increased risk.

The increase in breast cancer rates in recent decades has prompted researchers to explore the role of environmental factors in breast cancer etiology. One such factor is exposure to polychlorinated biphenyls (PCBs), which were manufactured in the United States for a variety of commercial uses from the 1930s until the late 1970s, when they were banned. These environmentally persistent compounds have exhibited endocrine system activity in the laboratory (Bonefeld-Jorgensen et al. 2001; Letcher et al. 2002; Oenga et al. 2004) and thus are of concern with respect to reproductive system effects, including breast cancer.

A systematic review found that total PCB exposure is not an important predictor for breast cancer in the general population (Negri et al. 2003). Some recent studies raise the possibility of differential genetic susceptibility, specifically with respect to *CYP1A1* polymorphisms (Li et al. 2004, 2005). If adverse human health effects from exposure to PCBs exist, they would most readily be identified in groups with the greatest exposures, such as worker populations.

Electrical capacitor production workers have been followed in several studies. Highly exposed workers (~ 2,500 individuals, including 1,318 females) in two plants that produced electrical capacitors in New York State and Massachusetts were studied by Brown and Jones (1981); the cohort was later updated by Brown (1987). The New York facility was included in mortality studies by Kimbrough et al. (1999, 2003). More recently, Prince et al. (2006b) expanded the cohort to include all workers at both facilities, regardless of exposure potential, in an update that employed a newly developed job-exposure matrix (JEM). Workers from a capacitor manufacturing plant located in Indiana were assessed in another retrospective cohort study (Sinks et al. 1992). An update of this study (Ruder et al. 2006) used a new JEM to estimate exposures. PCBs were used in capacitor production starting in 1939 at the Massachusetts plant, 1946 at the New York plant, and 1957 at the Indiana plant. The United States banned production of PCBs in 1977 (Smith and Brown 1987).

No previous study of these capacitor worker cohorts found large or significant elevations for female breast cancer mortality, and studies that examined exposure–response trends by duration (Prince et al. 2006a) or exposure level (Prince et al. 2006b) found no significant results. However, mortality is not a sensitive end point for outcomes with a high survival rate, such as breast cancer. Therefore, we decided to assess the relation between PCB exposure and female breast cancer incidence in a combined study of these three capacitor worker cohorts.

## Materials and Methods

The study population consisted of women included in prior retrospective cohort mortality studies of workers at three capacitor manufacturing plants in the United States. Plants 1 and 2, located in New York State and Massachusetts, respectively, employed a total of 13,321 women. Plant 3, located in Indiana, had 857 female employees. From the combined base cohort of 14,178 potentially PCB-exposed women, we restricted the present study to the 5,752 women who worked for at least 1 year at any of the three capacitor plants (41% of the total). The minimum employment restriction was motivated by the difficulty in locating women with short-term employment for questionnaire follow-up. Follow-up for breast cancer incidence began after 1 year of employment. This project received approval from the National Institute for Occupational Safety and Health (NIOSH) Human Subjects Review Board (HSRB). Per the HSRB-approved protocol, a returned questionnaire was considered implied informed consent. For study participants interviewed by telephone, we obtained oral informed consent before the interview.

### Questionnaires

We mailed a self-administered questionnaire to all women or their next of kin for whom we could determine valid addresses. After two mailings and a reminder postcard, we telephoned nonrespondents to obtain the information. We identified addresses and telephone numbers using sources such as the Internal Revenue Service (IRS), the U.S. Postal Service, motor vehicle registration records, credit bureaus, and telephone number lookup services. The questionnaire collected information on *a*) incident breast and other cancer diagnoses and details necessary to confirm diagnoses via medical records; *b*) relevant nonoccupational risk factors for breast cancer, such as family history and reproductive history; and *c*) demographic and lifestyle information such as ethnicity, education, height, weight, and smoking history. Because we knew that plant 2 included a number of workers of Cape Verdean origin, we offered Spanish- and Portuguese-language translations of the questionnaire to non-English-speaking respondents upon request. Questionnaires were collected from 1998 through 2000. Women reporting breast cancer diagnoses after 1998 were not considered cases.

Reliable information on race was generally not available from plant records. For many deceased workers, we obtained race information from death certificates and other vital status sources. The questionnaire actively sought race information, asking women to select from the following categories: white, black, American Indian or Alaskan Native, Asian or Pacific Islander, and other. Respondents selecting “other” for race were prompted to further specify their race. The questionnaire asked separately whether the worker was of Hispanic origin.

For the present study, we used race data from the plants and from death certificates only for calculating preliminary standardized incidence ratios (SIRs) and standardized rate ratios (SRRs). In these analyses, we assumed workers of unknown race to be white. We limited further analyses evaluating the effects of race to the subcohort with questionnaire data and used only the self- or proxy-reported data to classify workers by race. This approach differs from that used in previous studies of these cohorts (Prince et al. 2006a, 2006b; Ruder et al. 2006; Steenland et al. 2006), which used all data sources but prioritized questionnaire data. We opted to use only questionnaire-reported race for consistency and because of the potential for greater specificity.

### Breast cancer ascertainment

Vital status follow-up began in 1940 for the New York and Massachusetts facilities, and in 1957 for the Indiana plant, which opened in that year. Previous mortality studies of the cohorts (Brown and Jones 1981; Brown 1987; Prince et al. 2006a, 2006b; Ruder et al. 2006) used Social Security Administration (SSA), state motor vehicle registration, and state vital statistics offices to ascertain vital status in workers who died before 1979, and SSA, IRS, and National Death Index data for follow-up after this date. For all time periods, we used follow-back services such as the U.S. Postal Service and credit bureaus to complete ascertainment. We sought death certificates from state vital statistics offices and had underlying cause of death coded by trained nosologists according to the *International Classification of Diseases* (ICD) revision in effect at time of death (e.g., World Health Organization 1977).

For the present study, the end date for breast cancer incidence and mortality ascertainment was 31 December 1998. We identified additional breast cancer cases using questionnaires and cancer registries. Cancer registries were available in all three of the states in which the plants were located, but for varying periods of time (Massachusetts, 1982–1998; New York, 1976–1998; Indiana, 1987–1998). After we obtained the most current addresses, we evaluated the distribution by state of address and decided to also match our file against the Florida registry (covering 1981–1998) and the California registry (covering 1988–1998). Matching is done by staff at the registries, and data are not available to the public.

Questionnaire data were available for 201 of the 281 identified breast cancer cases (72%). If the report of breast cancer by a woman (or her next of kin) was specifically contradicted by another source of information (e.g., the medical record), we did not include the report as a case (*n* = 10). However, we included breast cancer cases reported in medical or cancer registry records (even if unreported by a woman or her next of kin) in the analysis (*n* = 82). We also included breast cancer cases for which we obtained no medical record. Medical record or cancer registry confirmation of the diagnosis was available for 43% and 55% of cases, respectively.

We obtained dates of breast cancer diagnosis from self-report, proxy report, the medical record, cancer registry record, or the death certificate. When diagnosis dates from multiple sources conflicted, we used the earliest date considered most valid (e.g., we considered medical record and cancer registry dates more valid than self-reported dates). For breast cancer decedents with only death certificate information (*n* = 10), we used the date of death as the date of diagnosis unless timing of diagnosis was otherwise noted on the death certificate, in which case we adjusted the date accordingly.

### Exposure estimates

Work history data from the three plants were collected by NIOSH in the mid-1970s. The plants began using PCBs at different times, but the overall exposure period for the cohort ranged from 1939 until 1977. We used plant-specific semiquantitative JEMs to allow ranking of exposure intensity for all workers across all three plants and across time. Sources used to develop the JEMs included work history records, a small number of air samples from the mid-1970s, information about historical process changes, job descriptions, and plant layouts. Separate JEMs for inhalation and dermal exposures in each plant used the same scale for exposure scores across plants and routes of exposure. We used an average of inhalation and dermal exposure scores to create the final plant-specific JEMs. These scores are unitless, because the dermal component could not be quantified; in the analysis, cumulative exposures are summed 1,000 unit-years. The exposure assessment process has been described in detail (Nilsen et al. 2004a, 2004b, 2005). We compared serum PCB levels from a sample of workers at two of the three plants with cumulative exposure scores calculated via the JEMs for the respective plants. Cumulative exposures estimated via the JEM correlated reasonably well with serum PCB levels (Steenland et al. 2006), with a higher correlation than that between duration of exposure and serum PCB level (Nilsen NB, Waters MA, Hein MJ, Ruder AM, unpublished data). Each plant had one department for which the exposure level could not be estimated but we believed to be low; for these departments, we used the lowest assigned estimate for the pertinent time period at that facility.

### Statistical analysis

We used the NIOSH Personal Computer Life Table Analysis System (PC-LTAS) to determine whether the incidence of breast cancer in the study cohort was higher than that expected from general population rates (Steenland et al. 1998). We constructed a referent rate file using data from the SEER (Surveillance, Epidemiology, and End Results) population for the period 1970–1999 (Ries et al. 2002), for invasive female breast cancer (ICD-9 code 174) and *in situ* breast cancer (ICD- 9 code 233.0).

Analyses using SEER referent rates produced SIRs and SRRs by categories of cumulative exposure and exposure duration, stratified by age (in 5-year categories), calendar time (in 5-year categories), and, for some analyses, race (white and nonwhite). Follow-up time began in 1970 when the SEER rates became available, or 1 year after employment, whichever was later. Beginning follow-up in 1970 eliminated 24 (8.5%) of the 281 breast cancer cases and 34.3% of the person-time from LTAS analyses only. Follow-up continued until the date of diagnosis, date of death, study end date (31 December 1998), or last known date before loss to follow-up, whichever was earliest. We also conducted lagged analyses to allow for the necessary latency period for solid tumors, with 0-, 10-, and 15-year lags examined. This lag discounts all exposure in the last years before the cutoff date, and sometimes results in a worker having no exposure or being “lagged out.” For exposure–response analyses, LTAS calculated a linear trend in a person-year–weighted regression of directly standardized rates, with statistical significance of each trend determined using a two-tailed *Z*-test with an **α** of 0.05.

We conducted internal exposure–response analyses using Cox regression. We used the SAS PHREG procedure (SAS Institute Inc. 2006), with age as the time variable (effectively matching on age). Risk sets for each case included all those who started working at a younger age than the index case’s age at diagnosis and who survived without breast cancer to at least that age. We truncated exposure at the age of the index case at diagnosis. In contrast with the SIR/SRR analyses, we retained lagged out workers in the regression analyses, with a zero dose assigned for the lagged out period.

We limited regression analyses for the full cohort (5,752 workers) to exploring exposure metrics and birth cohort effects. We considered several exposure metrics: cumulative exposure (log-transformed and untransformed), duration of exposure (log-transformed and untransformed), peak exposure, and average exposure (cumulative exposure divided by duration of exposure). We analyzed all metrics as continuous and categorical, with lags of 0, 10, and 15 years evaluated. We performed similar analyses for the subset of workers with some questionnaire data (the “questionnaire subcohort”) to see whether risks were similar in the two groups.

Because of the importance of certain covariates in assessing the relations between PCB exposure and breast cancer risk, we restricted further analyses to workers with complete questionnaire data on covariates of *a priori* interest (the “restricted subcohort”). We required members of the restricted subcohort (*n* = 3,141) to have data on parity, age at first live birth, replacement hormone use (ever and age at first use), whether a first-degree female relative had had breast cancer, and ever smoked at least 100 cigarettes. We assessed menopausal status but did not include it as a restricting factor; instead, we imputed the age of 52 for women for whom menopausal status or age at menopause was missing (*n* = 249).

We knew from prior studies (Brown 1987; Brown and Jones 1981; Prince et al. 2006a, 2006b; Ruder et al. 2006; Sinks et al. 1992) that the individual plants differed in exposure distribution, race, and percent hourly versus salaried, factors that could potentially affect breast cancer risk. Therefore, we evaluated the role of facility to determine whether further analyses should be restricted to individual facilities. In addition, because researchers have reported differences by ethnic background in PCB body burden in the vicinity of one of the plants (Choi et al. 2006), we evaluated race as a potential confounder warranting separate analyses. We also wanted to consider race in evaluating between facility differences, but only one facility (plant 2) had more than a single case among nonwhite workers. We also assessed the role of self- versus proxy-reported data.

We performed covariate analyses on the restricted cohort, using backward elimination to rule out the least influential of the potential confounders. We also examined key interaction terms, evaluating statistical significance with the chi-square likelihood ratio test.

## Results

Among the full cohort of 5,752 women, the mean ± SD duration of employment was 7.7 ± 4.5 years. A total of 1,413 (25%) had died by the study end date. We obtained completed questionnaires for 3,952 (69%) of the cohort, 80% from living respondents and 20% from next of kin of deceased cohort members. The primary reason for nonresponse was our inability to determine a correct address (21%). A total of 500 former workers and proxy respondents (8.7%) refused to complete the questionnaire, and 112 (2%) failed to respond after repeated attempts.

We found 281 incident breast cancer cases in the full cohort. Although mortality follow-up of the cohort was exhaustive, nonfatal incident cases occurring before initiation of the cancer registries or occurring in states other than Massachusetts, New York, Indiana, Florida, and California could be ascertained only via questionnaire. Approximately 20% of the cohort did not return a questionnaire and were not known to be deceased at end of follow-up; incident cases occurring outside the registry search may have been missed in this group, particularly in the early years of follow-up.

Of the full cohort, we identified 147 women by plant records and death certificates as being of races other than white. When we used the questionnaire responses instead, the number of workers (both cases and noncases) identified as being of races other than white rose to 282. This group includes workers identifying as members of specific races other than white (black, American Indian/Alaskan Native, Asian, or Pacific Islander), women who identified only as “other,” and women identifying as multiracial. Because the number of workers identifying as other than white was small, we kept this group together for all further analyses and refer to it herein as “nonwhite,” although some women in fact selected a multiracial identity where one of the races was white.

Of the 281 cases in the full cohort, we identified only eight as nonwhite from plant or death certificate data. However, among workers with complete questionnaire data on the covariates of *a priori* interest (restricted cohort), we identified 14 of the 145 cases as nonwhite. Twelve of these were from plant 2. Of the 268 noncase workers identified as nonwhite on the questionnaire, 67 identified as African American, and 201 identified as “other.” Most workers (*n* = 150) in the last group did not further specify their race/ethnicity; of those who did, 90% (*n* = 55) identified themselves solely as Cape Verdean or as Cape Verdean and some other ethnicity.

Table 1 provides selected demographic and exposure characteristics of the breast cancer cases and noncases in the entire cohort (*n* = 5,752) and the questionnaire subcohort (*n* = 3,952). In the entire cohort, on average, cases (*n* = 281) were born earlier than noncases and were exposed longer; these differences were highly statistically significant. Cases also had higher cumulative exposures. Breast cancer *in situ* was reported for 8 of the 281 cases (2.8%).

More data were available for the questionnaire subcohort, including demographic and lifestyle characteristics. Again, cases (*n* = 201) were born significantly earlier than noncases and were exposed longer. Cases had higher mean cumulative exposure than did noncases, but the difference was not statistically significant. Cases were significantly more likely to have a first-degree relative with breast cancer than were noncases. Average age at first live birth showed a slight but statistically significant difference. There were no statistically significant differences between case and noncases in the average age at menarche, body mass index (BMI), parity, number of children, use of hormone replacement therapy, age at first use of hormone therapy, or in the proportion of nonwhites or ever-smokers.

The plants differed in a number of ways. Most striking were the contrasts in exposure distributions. Mean ± SD estimated exposure for the workforce of plant 2 (0.80 ± 1.06, 1,000 unit-years) was more than twice that in plant 1 (0.34 ± 0.71) and was an order of magnitude greater than that in plant 3 (0.078 ± 0.10). The medians reflected a similar pattern (plant 2 = 0.39, plant 1 = 0.11, and plant 3 = 0.04). Although all three facilities had nonwhite populations < 4% according to records-based data, we classified > 15% of workers at plant 2 as nonwhite using questionnaire data. The percentages of nonwhite workers for plants 1 and 3 increased only slightly when we used questionnaire data.

In the aggregate, white and nonwhite workers in the restricted subcohort differed on a number of demographic and lifestyle factors (Table 2). Statistically significant differences included higher mean and median cumulative exposures, fewer pack-years of smoking, greater number of live births, and a greater percentage with a first-degree female relative with breast cancer among nonwhite workers. Mean exposure durations for the two groups were similar. We saw more striking differences among cases: The median exposure for nonwhite cases was nearly double the 75th percentile of exposure for white cases; in contrast, for noncases, the mean was only slightly higher in nonwhites and the median was actually slightly higher in whites.

SIR and SRR results reflect follow-up time and cases occurring in 1970 or later, because of availability of SEER comparison data. For the full cohort, the unlagged SIR was 0.81 [95% confidence interval (CI), 0.72–0.92; *n* = 257] and using a 10-year lag was 0.81 (95% CI, 0.71–0.92; *n* = 251 cases). SRRs by exposure level showed no trend using lagged or unlagged data. However, the SIRs and SRRs differed somewhat by race. For women identified as white by plant records or death certificates, the SIRs were 0.80 (95% CI, 0.70–0.90; *n* = 250) and 0.80 (95% CI, 0.70–0.90; *n* = 244) with 0- and 10-year lags, respectively. For women identified as nonwhite by records (all facilities), the SIRs had nonsignificant elevations of 1.87 (95% CI, 0.75–3.84; *n* = 7) and 1.94 (95% CI, 0.77–3.99; *n* = 7) for 0- and 10-year lags respectively. SIRs for women with questionnaire data were similar to those for the full cohort in white women (unlagged SIR = 0.84; 95% CI, 0.72–0.97; *n* = 176; 10-year lag SIR = 0.84; 95% CI, 0.72–0.97; *n* = 172) but showed a more modest elevation in nonwhite women (unlagged SIR = 1.14; 95% CI, 0.59–1.99; *n* = 12; 10-year lag SIR = 1.17; 95% CI, 0.06–2.03; *n* = 12) than that seen in the full cohort.

SRR results differed depending on dose cut points and lag (data not shown), particularly for white women, with no consistent patterns observed. In nonwhite women, trends were always positive and sometimes statistically significant, depending on cut points and lag. However, elevations were limited to the highest dose categories, with deficits observed in some intermediate categories.

Table 3 shows results of main effects exposure–response analyses via Cox regression using the full, questionnaire, and restricted cohorts. To account for secular trends in breast cancer incidence, all models included variables for birth cohort (before 1920 as a referent, 1920–1934, and ≥ 1935). Although the exposure metrics were right skewed, the untransformed metrics fit the data best. For the full cohort, neither of the exposure metrics achieved statistical significance. Cumulative exposure was not associated with elevations in breast cancer incidence for the full cohort, the questionnaire subcohort, or the restricted subcohort; exposure duration showed a statistically significant association with risk only in the restricted cohort. Results for external and internal analyses did not differ greatly with the inclusion or exclusion of *in situ* cases.

Because the SRR results suggested a difference in risk by race, we evaluated the effects of race in the regression analyses and then conducted further evaluations of potential confounders and effect modifiers in the restricted cohort separately for nonwhite and white women. Table 4 provides the results of these analyses. Although the unlagged model fit best for most exposure metrics (cumulative, duration) in white women, a 10-year lag fit better in nonwhite women. We had doubts about the biologic plausibility of a zero lag and chose the 10-year lag for further modeling.

Among white women, cumulative exposure and duration of exposure had little effect on breast cancer risk. Birth cohort was significant, with an increased risk for the group born during the period 1920–1934 and a larger increase compared with baseline for those born after 1934. Beyond birth cohort, the covariates retained in the model were parity, family history of breast cancer, and self- versus proxy questionnaire completion. Plant did not strongly affect the relation between the exposure metrics and breast cancer risk, and we eliminated this variable from the model. Use of hormone replacement therapy, although technically a confounder, had a lesser effect on risk, and we also eliminated this variable from the final model in the interest of parsimony. Menopausal status was not a confounder in this group.

In contrast, among nonwhite women, the effects of increasing exposure were positive and statistically significant. Both cumulative exposure (continuous) and duration of exposure were highly associated with breast cancer risk. Categorical exposure was associated with elevated risk at the two highest levels, but not in the intermediate category. The variables usually considered breast cancer risk factors (family history, parity, use of hormone replacement therapy, menopausal status) had little effect on risk in this group (data not shown). The most important covariates for this cohort were ever-smoking, source of questionnaire data, and birth cohort (limited to those women born after 1934). In univariate analyses, nonwhite cases smoked more than noncases (46 vs. 29 pack-years), in part because they smoked for more years (42.6 vs. 32.9). We did not observe these patterns among white workers. To assess whether smoking might be a proxy for alcohol use, we examined models with smoking but not alcohol, with alcohol but not smoking, and with both alcohol and smoking and found that only smoking had significant explanatory value.

To further explore the effects of smoking in nonwhite workers, we considered current smoking status (at time of death for deceased workers or time of questionnaire completion for living workers) and pack-years. Adding pack-years or current smoking status to a model that included ever smoking had little effect. The interaction between current smoking status and cumulative exposure nearly attained statistical significance (*p* = 0.06).

Because the mean and median PCB exposure estimates were significantly higher among nonwhite workers, we considered the possibility that the greater risk observed in nonwhite women was attributable to a high-exposure effect. We reevaluated the exposure risk in white women using the same cut points employed for nonwhite women and found the same lack of trend we observed with the original quartile-based cut points. The lack of exposure–response in white women does not appear to be related to the lower exposure levels in this group.

## Discussion

In this study of breast cancer incidence among female workers employed in capacitor production facilities, differences in risk by race, although subject to a number of caveats, were evident. In white women, the SIR was below expectation, and in the modeling analyses, the very small positive exposure response observed was largely explained by the well-known risk factors parity, age at first live birth, and family history of breast cancer. Differences between the SIR and exposure–response results likely resulted from differences in covariate control, in addition to differences between the subcohorts used in the SIR and regression analyses; despite these differences, we observed no statistically significant elevation of risk in any subcohort of white women. These results suggest that among white women, if there is a relation of PCB exposure with breast cancer risk, it is likely quite modest, even at the relatively high levels characteristic of occupationally exposed populations.

In contrast, among nonwhite women, whereas the SIR was close to expectation, the strong, positive exposure response seen in regression analyses was only modestly attenuated by smoking, birth cohort, and source of questionnaire data (self or proxy), the only covariates with significant explanatory power. Race was not a variable of strong *a priori* interest, and these findings may be the result of chance or uncontrolled confounding. The small number of nonwhite cases further limits interpretation of the findings in this group.

In both white and nonwhite women, risk of breast cancer after PCB exposure was lower for women born before 1920 than for those born after 1934. The pattern of increasing breast cancer incidence risk for later birth cohorts is generally consistent with results reported from the SEER registry (Holford et al. 2006) and the Connecticut Cancer Registry (Holford et al. 1991).

The study has several potential limitations. Response bias is a possibility when questionnaires are used. If nonrespondents had lower breast cancer rates than respondents, an artificial excess of breast cancer would be observed in the exposed population versus the nonexposed referent population. To minimize the likelihood of such bias, the study materials referred not to breast cancer but to “health effects.”

Of greater concern is that ascertainment of incident breast cancer cases was likely better for the later years of the study, when cancer registry data were available to supplement death records and questionnaire data. Although we identified only 10 breast cancer deaths using death certificates alone, it is likely that nonfatal incident cases were missed in the early years. This would lead to underestimates of the SIR and could affect exposure–response analyses, because early exposures were substantially higher than those in later years. In addition, this could affect observed secular trends. On the other hand, we could not verify some of the cases identified via questionnaire either by medical records or by a cancer registry, so we cannot rule out limited overascertainment.

Completion of questionnaires by proxy respondents likely introduced misclassification on some covariates. Proxy respondents often left blanks in response to some questions, so the restricted cohort underrepresents deceased workers. In addition, proxy respondents may also have been less accurate in responses to questions they did answer. When we performed a sensitivity analysis evaluating proxy and self-respondents in the restricted cohort separately, we found that results for each group were quite similar to those for all respondents combined, although risk estimates were slightly higher among self-respondents.

Errors in reporting of smoking are potentially problematic, given the results in nonwhite women. Studies have shown that smoking status is reported more accurately by proxies than was number of cigarettes smoked (Lerchen and Samet 1986; Steenland and Schnorr 1988). We used ever/never in the final model to minimize reporting bias and to avoid further restricting the cohort. The positive exposure response persisted whether we included or excluded smoking covariates.

Misclassification of PCB exposure is another potential limitation. We assessed exposure levels by job title, department, and work process, with limited air sampling data available from the 1970s only. The combination of dermal and inhalation exposures into a single semiquantitative estimate does not account for differences in doses by route of exposure. In addition, the use of total PCBs as the exposure metric may obscure the effects of individual congeners or subgroups.

Race, although an important predictor for breast cancer risk after PCB exposure in this study, is not well defined for this cohort. In addition to general concerns about using race in place of more specific information about genetics to explain disease risk (Harty et al. 2006; Kolonel et al. 2004), this study is hampered by marked discrepancies between race identified from records-based sources and race identified via questionnaire. In addition, whereas most nonwhite workers who more fully specified their race via questionnaire identified as either African American or Cape Verdean, more than half of the non-white workers self-identified as “other” and failed to further define their race. The potential for marked heterogeneity among those identifying as “other,” as well as the small number of nonwhite cases, cautions against overinterpretation.

Further specification of ethnic origins of all members of the cohort would be of interest because other studies raise the possibility that differences in nonoccupational exposure levels and/or genetic susceptibility could contribute to observed differences in risk by race. A study of women residing near a PCB-contaminated harbor in New Bedford, Massachusetts, close to where plant 2 is located, found higher PCB levels in infants of women from Portugal, the Azores, and Cape Verde compared with those born in the United States, Canada, or other countries (Choi et al. 2006). The authors considered the contributions of diet (i.e., fish consumption) but not occupational exposures. In addition, studies have found race-related differences in genetic polymorphisms that may affect PCB metabolism (Li et al. 2005), and slight elevations in breast cancer risk after PCB exposure in postmenopausal African-American women with *CYP1A1 M3* genotypes who smoked > 20 years (Li et al. 2004), suggesting the possibility of complex interactions between genes, lifestyle factors, and PCB-related breast cancer risk in a population subset.

Smoking is not generally considered a risk factor for breast cancer. The smoking results in the present study may represent a chance finding, or smoking may be a proxy for some other risk factor (although we ruled out alcohol, one obvious possibility) or a vehicle for greater but unmeasured PCB exposure through hand-to-mouth ingestion.

The lack of any significant overall increase in risk of breast cancer after PCB exposure in this occupational cohort is reassuring and consistent with the results of previous studies of the contributing cohorts (Prince et al. 2006a, 2006b; Ruder et al. 2006). Chance and residual confounding cannot be ruled out as explanations for the positive relation between PCB exposure and breast cancer risk among nonwhite women; however, this finding, which was not attenuated by reproductive factors, warrants further exploration.

## Figures and Tables

**Table 1:** Characteristics of breast cancer cases and noncases.

Variable	Cases	Noncases
Entire cohort		
No. of workers	281	5,471
Year of birth (mean ± SD)	1926 ± 11**	1930 ± 13
Years exposed (mean ± SD)	9.3 ± 8.2**	7.7 ± 7.6
Cumulative exposure (1,000 unit-years)		
Mean ± SD	0.67 ± 0.95	0.57 ± 0.93
Median	0.27*	0.22
Questionnaire subcohort^a^		
No. of workers	201	3,751
Year of birth (mean ± SD)	1928 ± 11**	1932 ± 12
Self-respondent on questionnaire (%)	63.7**	82.0
Nonwhite ethnicity (%)	7.5	9.3
Ever-smoker (%)	51.0	52.3
BMI at 20 years of age [kg/m^2^ (mean ± SD)]	20.8 ± 3.0	21.2 ± 3.2
Age at menarche [years (mean ± SD)]	12.8 ± 1.7	12.9 ± 1.7
Premenopausal at diagnosis (%)	15.4	NA
Parous (%)	86.1	86.7
No. of live births (mean ± SD)	2.5 ± 1.7	2.5 ± 1.8
Age at first live birth [years (mean ± SD)]	23.8 ± 4.2**	23.0 ± 4.5
Used hormone replacement therapy (%)	27.5	33.3
Age began hormone replacement use [years (mean ± SD)]	46.5 ± 8.8	47.2 ± 9.2
Breast cancer in first-degree female relative (%)	21.2*	13.9
Years exposed (mean ± SD)	8.9 ± 8.2*	7.4 ± 7.4
Cumulative exposure (1,000 unit-years)		
Mean ± SD	0.59 ± 0.84	0.52 ± 0.89
Median	0.27	0.20

NA, not applicable.

a Questionnaire subcohort comprises members of the full cohort for whom some questionnaire data were available. Not all participants (or their proxies) provided responses for each question; some variables have missing values.

* *p* < 0.05

** *p* < 0.01.

**Table 2:** Selected characteristics of restricted cohort^a^ by race.

Variable	White (*n* = 2,859)	Nonwhite (*n* = 282)
Cases and noncases		
Year of birth (mean ± SD)	1934 ± 11.6	1933 ± 10.6
Self-respondent on questionnaire (%)	90.0	89.0
Ever-smoker (%)	51.8	55.0
Pack-years (ever-smokers only) (mean ± SD)	35.2 ± 28.0	29.3 ± 24.0*
BMI at 20 years of age [kg/m^2^ (mean ± SD)]	21.2 ± 3.2	21.5 ± 3.6
Age at menarche [years (mean ± SD)]	12.9 ± 1.7	13.2 ± 1.9
Premenopausal at diagnosis (%)	19.2	14.3
Parous (%)	87.5	88.0
No. of live births (mean ± SD)	2.5 ± 1.7	2.9 ± 2.1**
Age at first live birth [years (mean ± SD)]	23.0 ± 4.4	22.5 ± 4.7
Used hormone replacement therapy (%)	33.2	31.6
Age began hormone replacement use [years (mean ± SD)]	47.3 ± 8.9	48.2 ± 10.4
Breast cancer in first-degree female relative (%)	13.5	18.8*
Years exposed (mean ± SD)	7.0 ± 7.1	6.6 ± 6.6
Cumulative exposure (1,000 unit-years)		
Mean ± SD	0.45 ± 0.81	0.64 ± 0.88**
Median	0.16	0.33**
Breast cancer cases only		
No. of cases	131	14
Cumulative exposure (1,000 unit-years)		
25th percentile	0.07	0.17
50th percentile	0.22	1.15
75th percentile	0.63	2.13

a Restricted cohort comprises women with questionnaire data for ever smoking, parity, age at first live birth, breast cancer in a first-degree female relative, hormone use, and age began hormone use. Some other variables have missing values.

* *p* < 0.05

** *p* < 0.01.

**Table 3:** Main effects exposure–response results for breast cancer incidence with a 10-year lag by subcohort: hazard ratio (95% CI).

Variable	Full cohort (*n* = 5,752, cases = 281)	Questionnaire subcohort^a^ (*n* = 3,952, cases = 201)	Restricted subcohort^b^ (*n* = 3,141, cases = 145)
Model 1			
Cumulative exposure (per 1,000 unit-years)	1.01 (0.96–1.05)	1.00 (0.94–1.06)	1.04 (0.97–1.11)
Born < 1920 (reference group)	1.00	1.00	1.00
Born 1920–1934	1.63 (1.19–2.23)	1.71 (1.14–2.57)	1.64 (0.96–2.83)
Born ≥ 1935	2.47 (1.65–3.70)	2.86 (1.74–4.71)	3.40 (1.79–6.46)
Model 2			
Years exposed	1.01 (0.99–1.03)	1.01 (0.99–1.03)	1.02 (1.00–1.05)
Born < 1920 (reference group)	1.00	1.00	1.00
Born 1920–1934	1.62 (1.18–2.22)	1.72 (1.14–2.58)	1.66 (0.96–2.86)
Born ≥ 1935	2.50 (1.67–3.75)	2.93 (1.78–4.84)	3.52 (1.85–6.72)

a Questionnaire subcohort comprises members of the full cohort for whom some questionnaire data were available.

b Restricted cohort comprises women with questionnaire data for ever smoking, parity, age at first live birth, breast cancer in first-degree female relative, hormone use, and age began hormone use.

**Table 4:** Full models for breast cancer incidence with a 10-year lag, restricted subcohort^a^ by race.

Variable	Hazard ratio (95% CI)
White women (131 cases)	
Model 1: Continuous cumulative exposure (per 1,000 unit*-*years)	1.00 (1.00–1.00)
Born < 1920 (reference group)	1.00
Born 1920–1934	3.07 (1.63–5.78)
Born ≥ 1935	7.81 (3.65–16.7)
Self-respondent	0.31 (0.19–0.50)
Parous	0.30 (0.10–0.84)
Age at first live birth	1.07 (1.03–1.11)
Positive family history	1.60 (1.05–2.44)
Model 2: Years exposed^b^	1.02 (0.99–1.05)
Model 3: Categorical cumulative exposure (per 1,000 unit*-*years)^b^	
0 to < 0.16 (*n* = 32)	1.00
0.16 to < 0.46 (*n* = 33)	1.36 (0.83–2.23)
0.46 to < 1.6 (*n* = 34)	1.15 (0.70–1.89)
≥ 1.6 (*n* = 32)	1.27 (0.75–2.17)
Nonwhite women (14 cases)	
Model 4: Continuous cumulative exposure (per 1,000 unit*-*years)	1.33 (1.14–1.56)
Born < 1920 (reference group)	1.00
Born 1920–1934	0.94 (0.22–4.07)
Born ≥ 1935	3.75 (0.51–27.8)
Self-respondent	0.07 (0.02–0.26)
Ever-smoker	3.84 (1.01–14.6)
Model 5: Years exposed^c^	1.13 (1.03–1.23)
Model 6: Categorical cumulative exposure (per 1,000 unit*-*years)^c^	
< 0.47 (*n* = 4)	1.00
0.47 to < 3.9 (*n* = 3)	0.60 (0.12–3.01)
3.9 to < 5.8 (*n* = 4)	7.65 (1.11–52.8)
≥ 5.8 (*n* = 3)	22.3 (2.38–209)

a Restricted cohort comprises women with questionnaire data for ever smoking, parity, age at first live birth, breast cancer in first-degree female relative, hormone use, and age began hormone use.

b Models control for birth cohort (1920–1934, ≥ 1935, vs. < 1920); self-respondent, parity, age at first live birth, and positive family history).

c Models control for birth cohort (1920–1934, ≥ 1935, vs. <1920); self-respondent and ever-smoker).
